# Intratumoral and peritumoral radiomics using multi-phase contrast-enhanced CT for diagnosis of renal oncocytoma and chromophobe renal cell carcinoma: a multicenter retrospective study

**DOI:** 10.3389/fonc.2025.1501084

**Published:** 2025-02-05

**Authors:** Yongsong Ye, Bei Weng, Yan Guo, Lesheng Huang, Shanghuang Xie, Guimian Zhong, Wenhui Feng, Wenxiang Lin, Zhixuan Song, Huanjun Wang, Tianzhu Liu

**Affiliations:** ^1^ Department of Radiology, Guangdong Provincial Hospital of Traditional Chinese Medicine, Guangzhou, China; ^2^ Department of Radiology, The First Affiliated Hospital, Sun Yat-Sen University, Guangzhou, China; ^3^ Department of Radiology, Guangdong Provincial Hospital of Traditional Chinese Medicine, Zhuhai, China; ^4^ Lab of Molecular Imaging and Medical Intelligence, Department of Radiology, Longgang Central Hospital of Shenzhen, Shenzhen Clinical Medical College, Guangzhou University of Chinese Medicine, Longgang Central Hospital of Shantou University Medical College, Shenzhen, China; ^5^ Department of Radiology, The Affiliated Panyu Central Hospital of Guangzhou Medical University, Guangzhou, China; ^6^ Department of Radiology, Zhuhai People’s Hospital, Zhuhai, China; ^7^ Department of Radiology, The First Affiliated Hospital of GuangZhou Medical University, HengQin Hospital, Zhuhai, China; ^8^ Clinical and Technical Support, Philips Healthcare, Guangzhou, China

**Keywords:** renal oncocytoma, chromophobe renal cell carcinoma, peritumoral, intratumoral, radiomics

## Abstract

**Purpose:**

To construct diagnostic models that distinguish renal oncocytoma (RO) from chromophobe renal cell carcinoma (CRCC) using intratumoral and peritumoral radiomic features from the corticomedullary phase (CMP) and nephrographic phase (NP) of computed tomography, and compare model results with manual and radiological results.

**Methods:**

The RO and CRCC cases from five centers were split into a training set (70%) and a validation set (30%). CMP and NP intratumoral and peritumoral (1–3 mm) radiomic features were extracted. Segmentation was performed by radiologists and software. Features with high intraclass correlation coefficients (ICC>0.75) were selected through univariate analysis, followed by the LASSO method to determine the final features for the SVM model. All images were assessed by two radiologists, and radiological reports were also examined. The diagnostic performances of the different methods were compared using several statistical methods.

**Results:**

The training set had 65 cases (29 RO, 36 CRCC) and the validation set had 27 cases (12 RO, 15 CRCC). All the training models had excellent performance (area under the curve [AUC]: 0.828–0.942); the AUC values of the validation models ranged from 0.900 (Model 4) to 0.600 (Model 2). CMP models (AUC: 0.811–0.900) generally outperformed NP and fusion models (AUC: 0.728–0.756). SVM models (sensitivity: 62.50–88.89%; specificity: 63.16–77.78%; accuracy: 62.96–81.48%) outperformed manual diagnosis (sensitivity: 46.74–70.59%; specificity: 41.67–46.34%; accuracy: 52.27–59.78%). The clinical reports alone had no diagnostic value.

**Conclusion:**

CMP intratumoral and peritumoral radiomics models reliably distinguished RO from CRCC.

## Introduction

Renal oncocytoma (RO) is a benign eosinophilic kidney tumor that accounts for 3 to 7% of all renal neoplasms (1). Due to its indolent nature, surgical resection of RO is often unnecessary (2), and nephron-sparing surgery is considered the standard treatment; other treatment options are cryoablation, radiofrequency ablation, high-intensity focused ultrasound, microwave thermotherapy, and interstitial photon irradiation (3, 4). However, radiological differentiation of RO from malignant renal tumors, particularly chromophobe renal cell carcinoma (CRCC, which may require nephrectomy and has a potential risk of metastasis (5)) can be very challenging (2).

Radiomics has significant potential for classifying the nature of lesions (6). This quantitative approach analyzes subtle but distinctive characteristics of medical images, and can provide a better understanding and identification of different tumor phenotypes (7, 8). Previous research demonstrated the value of radiomics in studies of kidney neoplasms, suggesting this approach may also be useful in clinical settings (9–11), including the differentiation of RO and CRCC (12–16). However, previous radiomics studies have primarily focused on intratumoral features, and applications to clinical settings and comparisons with outcomes predicted by manual diagnosis have been limited. Notably, there is evidence that a radiomics approach that also considers the peritumoral region can significantly improve the performance of diagnostic models (17, 18). Furthermore, the explicit comparison of different radiomics models with manual diagnosis may provide a more intuitive and convincing assessment of model performance (9, 10).

This study aimed to develop diagnostic models for distinguishing RO from CRCC using the corticomedullary phase (CMP) and nephrographic phase (NP) of intratumoral and peritumoral computed tomography (CT)-based radiomics, and to compare the results with those from manual diagnoses and radiology reports.

## Methods

### Ethical approval and collection of cases

This multicenter retrospective study was approved by the Medical Ethics Committee of Guangdong Provincial Hospital of Chinese Medicine (Approval No: ZE2024-294), which waived the need for written informed consent. Cases of RO and CRCC were identified from the pathology reporting systems and Picture Archiving and Communication Systems (PACS) of five centers in Guangdong Province: Guangdong Hospital of Traditional Chinese Medicine, Guangzhou (Center 1); Guangdong Hospital of Traditional Chinese Medicine, Zhuhai (Center 2); The First Affiliated Hospital of Sun Yat-sen University, Guangzhou (Center 3); The Affiliated Panyu Central Hospital of Guangdong Medical University, Guangzhou (Center 4); and Longgang Central Hospital, Shenzhen (Center 5). The study period was from January 2018 to July 2024.

**Figure 1:**
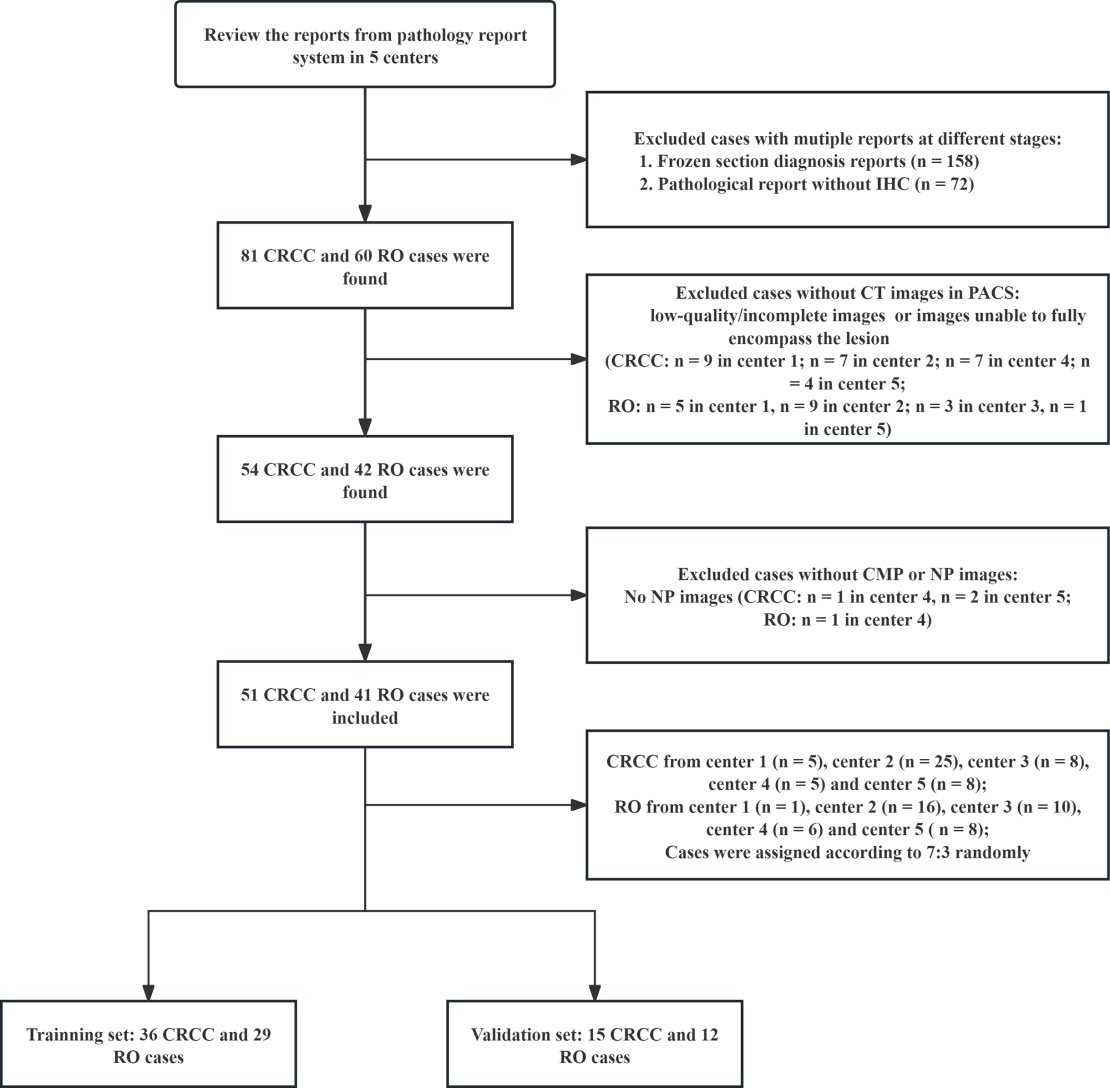
Patient disposition and establishment of the training set and validation set.

The inclusion criteria were availability of (a) enhanced CT scans, including images from the CMP and NP; (b) complete clinical data, such as age, sex, lesion location, comprehensive operation (including radical nephrectomy or local nephrectomy), and histopathological and immunohistochemical findings from each center’s pathology records; and (c) intact CT images stored in the PACS. The exclusion criteria were: (a) low-quality or incomplete CT images; and (b) CT scans that did not fully encompass the lesion. Application of these criteria led to identification of 92 eligible patients, 51 with CRCC and 41 with RO (**Figure 1**).

### Histological and immunohistochemical diagnosis

All diagnoses were confirmed by hematoxylin-eosin (HE) staining and immunohistochemistry (IHC) after tumor excision using standard guidelines (19). The histopathological criteria for CRCC were: pale or clear cytoplasm, distinct perinuclear halo, and large, heterogeneous nuclei. The histopathological criteria for RO were: eosinophilic cytoplasm, uniform nuclear morphology, and pale brown solid or nodular structures. The immunohistochemical diagnosis required staining for at least 2 of the 3 following specific markers: CD117 (positive in CRCC and negative in eosinophilic tumors), CK7 (positive in CRCC and negative or weakly positive in eosinophilic tumors), and S-100A1 (negative in CRCC and positive in eosinophilic tumors). Each diagnosis was reviewed and confirmed by at least two pathologists.

### Computed tomography

All included cases received unenhanced and dual-phase contrast-enhanced CT. These CT scans were performed using five different scanners: Definition Flash (Siemens, Germany) and IQon Spectral (Philips Healthcare, Netherlands) at Center 1; Aquilion One 750 W (Canon, Japan) at Center 2; IQon Spectral (Philips Healthcare, Netherlands) at Center 3; APEX (GE Healthcare, USA) at Center 4; and Revolution (GE Healthcare, USA) at Center 5. The scanning parameters were consistent across all centers, including tube voltage (100–140 kV), tube current (100–250 mA), section interval and thickness (1–5 mm), and matrix size (512 × 512 mm). Following an unenhanced scan, 100 to 120 mL of contrast medium (Ultravist 370, Bayer Schering Pharma, Germany at Centers 1 and 2; Ioversol 350, Hengrui Medicine, China at Centers 3 and 4; and iohexol 350, Fuan Pharmaceutical Group, China at Center 5) was injected at a flow rate of 3 to 4 mL/s. The CMP was scanned using an aortic monitoring trigger, and the NP was scanned after a 60 to 70 s delay. Two experienced radiologists (S.H.X and G.M.Z, with 17 and 18 years of experience, respectively) analyzed all CT images to ensure they met the inclusion criteria.

### Image segmentation

Image segmentation was performed using the open-source ITK-SNAP software (http://www.itksnap.org) by two radiologists with experience in abdominal radiography (T.Z.L and L.S.H, with more than 15 and 17 years of experience, respectively). The entire tumor mass was meticulously segmented on the original CMP and NP CT images to avoid over- or under-segmentation. Each radiologist independently segmented all images. To ensure consistency, images and masks were resampled to a voxel size of 1 × 1 × 1 mm³. Using the ‘scipy.ndimage’ package in Python, the peritumoral regions were dilated to 1 mm, 2 mm, and 3 mm beyond the tumor boundaries, and the resulting masks were saved for further analysis. The ‘PyRadiomics’ package in Python was then used to extract 2260 intratumoral and 6780 peritumoral radiomic features from the CMP and NP images. These features were: shape, texture, first-order statistics, Laplacian of Gaussian (LoG), Gray-Level Co-occurrence Matrix (GLCM), Gray-Level Run-Length Matrix (GLRLM), Gray-Level Size Zone Matrix (GLSZM), Neighboring Gray Tone Difference Matrix (NGTDM), and Gray Level Dependence Matrix (GLDM). These features were used with 14 additional filters, including exponential, gradient, square, and wavelet transforms. All features were normalized using *z*-score standardization.

To assess the stability of the radiomic features, 40 cases were randomly selected, and their tumors were re-segmented by radiologists (S.H.X and G.M.Z) to evaluate intra- and inter-reader reliability based on the intraclass correlation coefficient (ICC). Any feature with an ICC greater than 0.75 was deemed stable and included in the analysis. A two-sample *t*-test was used to identify potentially significant radiomic features, and the least absolute shrinkage and selection operator (LASSO) method was then used to select the most appropriate features. LASSO was applied with 10-fold cross-validation to determine the optimal regularization parameter (λ) (17). Coefficients for each radiomic feature were calculated, and only those with non-zero coefficients were retained for further analysis, ensuring the most relevant features were included in the final model.

### Model development

**Figure 2:**
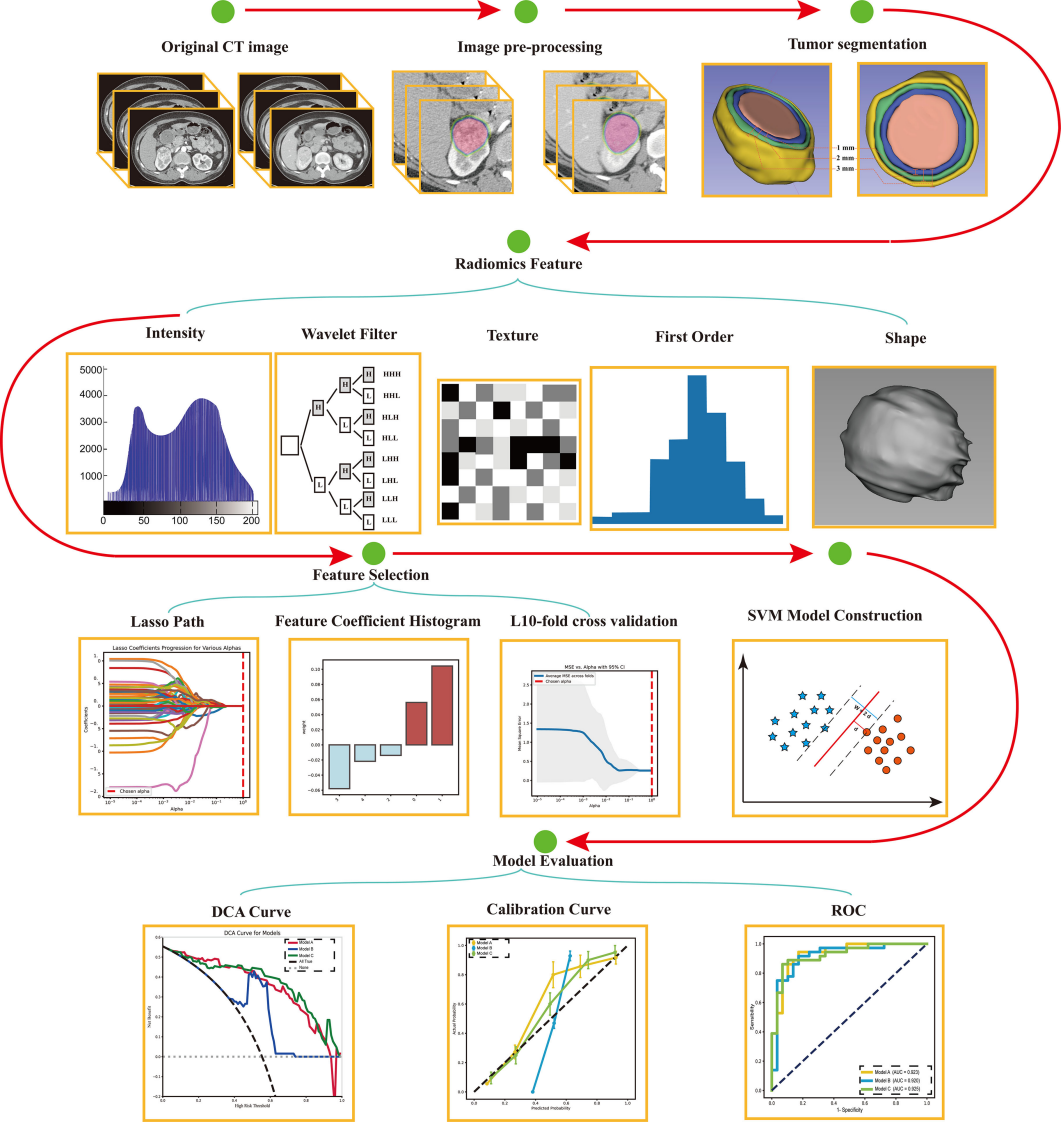
Workflow used to develop and evaluate the support vector machine classifiers.

Nine types of radiomic models were developed: Model 1, CMP intratumoral model; Model 2, CMP intratumoral + CMP 1 mm peritumoral model; Model 3, CMP intratumoral + CMP 1 mm & 2 mm peritumoral model; Model 4, CMP intratumoral + CMP 1 mm, 2 mm & 3 mm peritumoral model; Model 5, NP intratumoral model; Model 6, NP intratumoral + NP 1 mm peritumoral model; Model 7, NP intratumoral + NP 1 mm & 2 mm peritumoral model; Model 8, NP intratumoral + NP 1 mm, 2 mm & 3 mm peritumoral model; and Model 9, CMP intratumoral + CMP 1 mm, 2 mm & 3 mm + NP intratumoral + NP 1 mm, 2 mm & 3 mm peritumoral model. Support vector machine (SVM) models, which are widely used in radiomics, were used for model classification. A 10-fold cross-validation strategy was employed, where 9 parts were used for training and 1 part was used for validation. A grid search method was applied to optimize the hyperparameters. The cases were shuffled and then divided into a training set (36 CRCC and 29 RO cases) and a validation set (15 CRCC and 12 oncocytoma cases) in a 7:3 ratio. The workflow for the radiomic approach is illustrated in **Figure 2**.

### Manual diagnosis by radiologists

The diagnostic performances of two radiologists (W.H.F. and W.X.W, with over 8 and 7 years of experience, respectively) were also evaluated. These radiologists were not affiliated with the study centers and were blinded to patient demographics, clinical characteristics, and histopathologic results. They used the open-source DICOM viewer, MicroDicom (https://www.microdicom.com/), to evaluate the unenhanced, CMP, and NP CT images and provided diagnoses (benign or malignant). One radiologist (W.H.F) also noted the maximum tumor diameter, morphology (regular or irregular), margin clarity, necrosis (present or absent), enhancement pattern (uniform or uneven), and invasion of surrounding structures. To further evaluate diagnostic efficacy, CT radiology reports were collected, and the findings were categorized as benign or malignant. If a report suggested the possibility of malignancy, it was classified as malignant, even if the possibility of benign was also mentioned.

### Statistical analysis

Statistical analyses were conducted using SPSS version 23.0 and Python version 3.7.1. Python was used for feature extraction, screening, and model development and validation, and SPSS was used for comparative analysis between cohorts. All statistical tests were two-sided, and a P-value less than 0.05 was considered significant. Continuous variables are reported as mean ± standard deviation (SD), and categorical variables as frequency and percentage. The χ^2^ test was used to compare categorical data, and the independent-samples *t*-test or Wilcoxon test was used to compare clinical data between groups. The ICC was used to assess the consistency of radiomic features extracted by different radiologists.

The discriminative performance of each model was evaluated by receiver operating characteristic (ROC) analysis, with calculation of area under the curve (AUC), accuracy (ACC), sensitivity (SENS), specificity (SPEC), positive predictive value (PPV), and negative predictive value (NPV). The AUCs of the different radiomic models were compared using the DeLong test. The importance weight of models structured by CMP, NP, and fusion radiomic features (model 4, model 7, and model 9) were analyzed using the ‘sklearn.ensemble’ package in Python. The ‘sklearn.calibration’ package in Python was also used with custom code to plot calibration curves, for decision curve analysis (DCA), and to present the SENS, SPEC, ACC, PPV, NPV, and 95% confidence intervals (CIs) obtained from ROC analysis.

## Results

### Patient characteristics

This study included 51 patients with CRCC and 41 patients with RO, which we divided into a training set (n = 65) and a validation set (n = 27) in a 7:3 ratio (**Table 1**). These two groups had no significant differences in age, sex, operation type, or tumor location.

**Table 1:** Characteristics of CRCC and RO patients in the training set and validation set^*^.

Characteristic	Training set (n = 65)			Validation set (n = 27)		
	CRCC (n = 36)	RO (n = 29)	*P* value	CRCC (n = 15)	RO (n = 12)	*P* value
Age (years), mean ± SD	56.4 ± 15.7	51.4 ± 13.63	0.10	51.4 ± 13.63	54.5 ± 8.96	0.69
Gender, n (%)						
Male	16 (17.58%)	11 (12.09%)	0.39	6 (6.60%)	5 (5.50%)	0.93
Female	20 (21.98%)	18 (19.78%)		9 (9.89%)	7 (7.69%)	
Location, n (%)						
Right kidney	17 (18.68%)	16 (17.58%)	0.62	8 (8.79%)	5 (5.50%)	0.42
Left kidney	19 (20.88%)	13 (14.29%)		7 (7.69%)	7 (7.69%)	
Operation type						
Radical nephrectomy	17	8	0.10	10	4	0.06
Local nephrectomy	19	20		5	9	

*Data are expressed as mean ± standard deviation (SD) or n (%). CRCC, chromophobe renal cell carcinoma; RO, renal oncocytoma.

### Selection of radiomic features

We selected radiomic features from the CMP and NP CT images using the LASSO method with 10-fold cross-validation (**Supplementary Table S1**). The number of features in each model ranged from 3 (Model 5) to 33 (Model 9). We excluded models 6 and 8 because they had no significant radiomic features in the 1 mm or 3 mm peritumoral regions of the NP images.

### Diagnostic performance of machine learning algorithms

**Figure 3:**
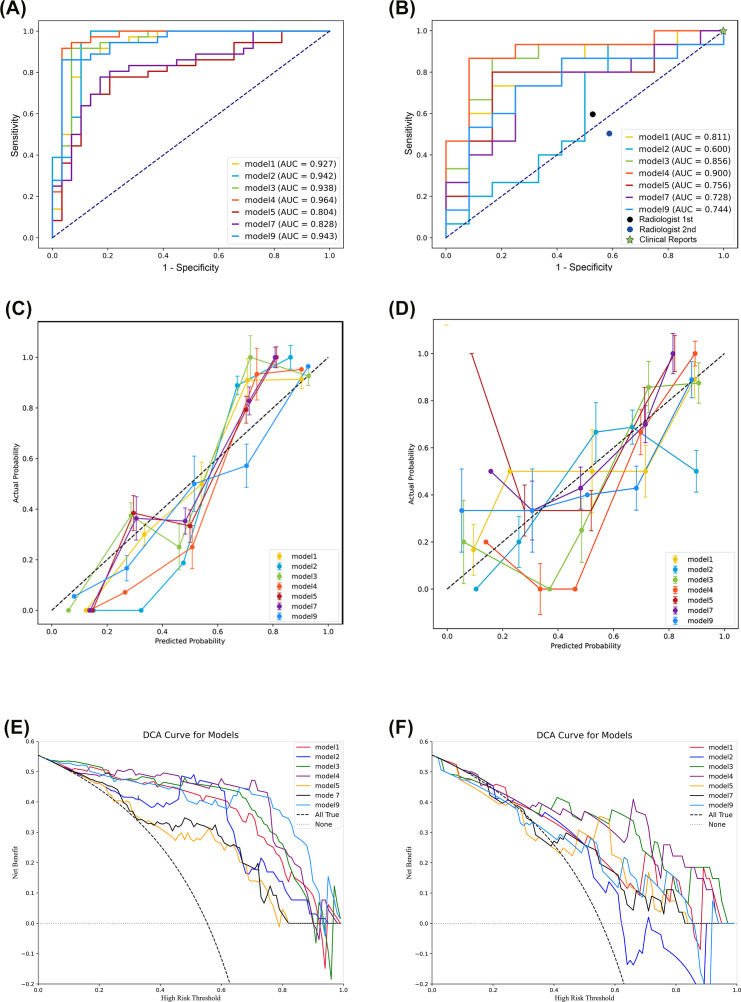
Evaluation of the support vector machine classifiers based on receiver operating characteristic analysis **(A, B)**, calibration curves **(C, D)**, and decision curve analysis **(E, F)** of the training set (left) and the validation set (right).

We then developed machine learning models using each of the seven models that had significant radiomic features, and evaluated their diagnostic performance using ROC analysis for the training set and validation set (**Figures 3A, B**, **Table 2**). In the training set, the SVM classifiers for Model 1 (SENS: 88.89%; SPEC: 86.84%; ACC: 87.69%; AUC: 0.927), Model 2 (SENS: 91.89%; SPEC: 92.86; ACC: 92.30%; AUC: 0.942), Model 3 (SENS: 89.29%; SPEC: 89.19%; ACC: 89.23%; AUC: 0.938), Model 4 (SENS: 92.59%; SPEC: 89.47%; ACC: 90.77%; AUC: 0.964), and Model 9 (SENS: 88.46%; SPEC: 84.62%; ACC: 86.15%; AUC: 0.942) demonstrated satisfactory and comparable diagnostic performance, and slightly poorer performance for Model 5 (SENS: 72.00%; SPEC: 72.50%; ACC: 72.31%; AUC: 0.804) and Model 7 (SENS: 75.86%; SPEC: 80.56%; ACC: 86.15%; AUC: 0.828).

**Table 2:** Differentiation of RO from CRCC by SVM classifiers of radiomic features from the CMP and NP phases of CT in the training set and validation set; by two independent radiologists; and by clinical reports.

Machine learning algorithm /Manual analysis	Sensitivity (%), (95% CI)	Specificity (%), (95% CI)	Accuracy (%), (95% CI)	PPV (%), (95% CI)	NPV (%), (95% CI)	AUC (95% CI)
Training set						
Model 1	88.89(78.57-96.55)	86.84(81.08-97.22)	87.69(96.92-80.00)	82.76(75.86-96.55)	91.67(83.33-97.22)	0.927(0.846-1.00)
Model 2	91.89(85.71-98.07)	92.86(82.76-100)	92.30(83.08-99.60)	89.66(85.76-93.56)	94.44(86.11-100)	0.942(0.891-0.992)
Model 3	89.29(85.71-96.42)	89.19(86.11-94.59)	89.23(86.15-95.38)	86.20(82.76-93.10)	86.67(80.00-93.33)	0.938(0.843-1.00)
Model 4	92.59(85.74-100)	89.47(84.21-100)	90.77(83.08-100)	86.21(79.31-93.10)	93.33(90.00-96.67)	0.964(0.886-1.00)
Model 5	72.00(65.22-80.65)	72.50(66.66-80.56)	72.31(66.16-84.62)	62.07(55.17-75.86)	80.56(77.78-83.33)	0.804(0.752-0.855)
Model 7	75.86(67.74-84.00)	80.56(74.29-86.85)	78.46(73.85-81.54)	75.86(72.41-79.31)	80.56(77.78-83.33)	0.828(0.775-0.880)
Model 9	88.46(82.76-92.59)	84.62(78.32-90.92)	86.15(84.61-89.23)	79.31(74.12-84.50)	91.67(86.11-97.22)	0.942(0.851-1.00)
Validation set						
Model 1	75.00(66.67-77.78)	68.42(66.67-75.00)	70.37(66.67-74.07)	50.00(43.00-66.67)	86.67(80.00-93.34)	0.811(0.683-0.940)
Model 2	62.50(53.57-71.43)	63.16(50.59-70.59)	62.96(55.55-70.37)	41.67(25.01-58.33)	80.00(73.33-86.67)	0.600(0.518-0.682)
Model 3	80.00(70.00-90.91)	76.47(68.75-87.50)	77.78(66.67-88.88)	86.21(82.76-93.10)	86.67(80.00-93.33)	0.856(0.731-0.980)
Model 4	88.89(83.33-94.54)	77.78(73.68-82.35)	81.48(74.07-85.19)	66.67(58.33-75.00)	93.33(80.00-100.0)	0.900(0.792-1.00)
Model 5	72.73(66.67-78.79)	72.50(66.67-80.56)	74.07(66.67-81.47)	66.67(58.33-75.00)	80.00(66.67-93.33)	0.756(0.674-0.837)
Model 7	64.29(60.00-69.23)	76.92(71.43-82.41)	70.37(66.67-74.07)	75.00(66.67-83.33)	66.67(60.00-73.33)	0.728(0.644-0.812)
Model 9	71.43(66.67-76.92)	65.00(57.77-69.23)	66.67(62.96-70.37)	41.67(25.01-58.33)	86.67(80.00-93.33)	0.744(0.634-0.855)
Radiologist-1	70.59	46.34	59.78	62.07	55.88	N/A
Radiologist-2	46.74	41.67	52.27	45.10	48.78	N/A
Clinical Reports	100	0	56.04	56.04	0	N/A

CMP, corticomedullary phase; NP, nephrographic phase; CI, confidence interval; PPV, positive predictive value; NPV, negative predictive value; AUC, area under the curve; SVM, Support Vector Machine; CRCC, chromophobe cell carcinoma.

In the validation set, analysis of models derived from CMP radiomic features showed that Model 1 (SENS: 75.00%; SPEC: 68.42%; ACC: 70.37%; AUC: 0.811), Model 3 (SENS: 80.00%; SPEC: 76.47%; ACC: 77.78%; AUC: 0.856), and Model 4 (SENS: 88.89%; SPEC: 77.78%; ACC: 81.48%; AUC: 0.900) outperformed Model 2 (SENS:62.50%; SPEC:63.16%; ACC:62.96%; AUC:0.600), and that Model 4 was the best overall. Even after incorporating the 2 mm NP peritumoral radiomic features, Model 7 (SENS: 64.29%; SPEC: 76.92%; ACC: 70.37%; AUC: 0.728) did not outperform Model 4. Similarly, Model 9 (SENS: 71.43%; SPEC: 65.00%; ACC: 66.67%; AUC: 0.744), which combined CMP and NP radiomic features, had poorer diagnostic performance than Models 1, 3, and 4, and was similar to Model 5 (SENS: 72.73%; SPEC: 72.50%; ACC: 74.07%; AUC: 0.756). Pairwise comparisons using the DeLong test indicated significant differences between Model 2 and Models 1, 3, and 4; and significant differences between Model 9 and Models 3 and 4 (**Supplementary Table S2**). The importance weight of radiomics features in model 4, model 7 and model 9 (**Supplementary Figure S1**) showed that the CMP features had the largest absolute weight in fusion models.

We also performed calibration curve analysis and DCA for the SVM classifiers in the training and validation sets. The calibration curves were relatively close to the ideal line in the training set (**Figure 3C**), but had several notable deviations in the validation set (**Figure 3D**). Specifically, the line for Model 2 deviated downward in the last third, and the lines for Models 5, 7, and 9 deviated upward in the first third. The DCA indicated excellent performance in the training set for Models 1, 2, 3, 4, and 9, but poor performance for Models 5 and 7 (**Figure 3E**). DCA of the validation set showed that Models 2, 5, and 7 had poorer performance than Models 1, 3, 4, and 9 (**Figure 3F**).

### Diagnostic performance of two radiologists and clinical reports

We also assessed the performance of manual diagnosis by the two radiologists and the clinical reports (**Tables 2**, **3**). The radiologists had accuracies of 59.78% and 46.74%, sensitivities of 70.59% and 52.27%, and specificities of 46.34% and 41.67%. There were statistically significant differences in tumor size, morphology, and CMP enhancement patterns between RO and CRCC. The clinical radiology reports aligned with the predefined criteria, in that all RO cases were misdiagnosed and all CRCC cases were correctly diagnosed as malignant. In other words, all 92 cases were reported as malignant, even though 9 reports (4 for RO and 5 for CRCC) suggested the possibility of benign lesions.

**Table 3:** Characteristics of the CRCC and RO cases determined by visual inspection of two radiologists^*^.

Characteristics	CRCC (n = 51)	RO (n = 41)	*P* value
Maximum diameter	31.64 (23.33, 42.19)	45.53 (31.53, 67.85)	<0.01
Morphology (regular/irregular)	39/12	38/3	0.04
Necrosis (no/yes)	41/10	36/5	0.34
CMP enhancement pattern (uniform/uneven)	23/28	27/14	<0.01
NP enhancement pattern (uniform/uneven)	17/34	16/25	0.57
Invasion of surrounding structures (yes/no)	51/0	41/0	N/A

*Data are expressed as median (interquartile range) or n/n.

CRCC, chromophobe renal cell carcinoma; RO, Renal oncocytoma.

## Discussion

This multicenter study explored the use of intratumoral and peritumoral radiomic features based on the CMP and NP of CT images to differentiate CRCC from RO. More specifically, we compared the diagnostic performance of multiple SVM classifiers with that of experienced radiologists. The two major findings were: (*i*) most of the radiomic models had excellent diagnostic performance and often outperformed the radiologists and (*ii*) models that used CMP features generally outperformed models using NP features and models that combined both features.

The accurate preoperative diagnosis of RO and CRCC remains a significant challenge in clinical practice. Li et al. (20) and Zhou et al. (21) studied these two cancers and developed models based on CT features that had promising diagnostic efficiency (AUC: 0.923 and 0.888, respectively). Akın et al. (22) also achieved excellent diagnostic discrimination of these cancers using MRI, particularly in the NP (AUC: 0.881) and the excretory phase (AUC: 0.900). Although we also identified statistically significant differences in the visual characteristics of these tumors (20, 23) (**Table 3**), applying these findings in clinical practice is challenging because these cancers often lack obvious visual distinctions, and radiologists may be biased toward the more cautious diagnosis of CRCC in official reports due to medico-legal considerations.

Compared to traditional visual assessment of CT images, a radiomics approach offers a more detailed and quantitative analysis of tumor heterogeneity, and this may provide a more accurate characterization of lesion pathology (9, 24, 25). This study builds upon previous research, in that it validated the combined use of intratumoral and peritumoral radiomic features for the discrimination of RO from CRCC. Although previous studies (1, 12, 13, 15) also demonstrated the efficacy of intratumoral radiomic features for discrimination of RO from CRCC, the present study is the first to integrate intratumoral and peritumoral features in CT-based radiomic models and then compare the diagnostic performance of these models with that of experienced radiologists and clinical radiology reports.

Previous research also reported variability in diagnostic performance among models that consider different peritumoral regions (26). The poorer performance of our Model 2 might be attributed to the smaller number of pixels within the region of interest (ROI), leading to less stable and less representative radiomic features (27). Conversely, our Models 3 and 4, which incorporated larger peritumoral regions, showed excellent diagnostic performance, and this points to the importance of having a sufficient pixel density in the ROI. The superior performance of CMP-based models compared to NP-based models and NP-CMP fusion models suggests that CMP-derived radiomic features alone appear to be sufficient for developing reliable diagnostic models, and this could also reduce computational demands. Although this result contrasts with some previous findings (14), the multicenter design and use of appropriate sample ratios in our study increased the stability and generalizability of the results. On the other hand, whether based on the diagnostic radiomics of RO, CRCC (14, 28), and other tumors that originate in the kidneys (29, 30), the CMP model has more texture and non-texture features with diagnostic significance than the NP model, and this is the reason for the superior diagnostic performance of the CMP model. Nguyen et al. (28) suggested that the CMP and NP can both detect differences in the nature of renal parenchymal space-occupying lesions; however, whether based on subjective observations, objective measurements, or screening of radiomics features, CMP provide more clinically meaningful information. The key reason for this might be differences in the enhancement of renal masses; CMP provides the greatest enhancement, and can show the key features and biological characteristics of tumors, and this is followed by gradual washout and decreased resolution of features. Similarly, we speculate that peritumoral radiomics features may also be affected by the CT scanning phase. Although there have been few relevant studies of this in patients with OR and CRCC, this was finding was reported in studies of other tumors (31). Additionally, the frequent use of LoG and wavelet features in these models, coupled with their significant weight in models, aligns with previous studies (14), and suggests their importance in differentiating RO from CRCC.

During the selection and screening of features, we employed several important considerations. Our original intent was to construct models based on deep-learning features and compare them with traditional radiomics features. Deep-learning features can automatically capture high-level semantic information (32, 33), but typically require large annotated datasets to prevent overfitting. Due to the limited sample size in this study, we were unable to utilize deep-learning features. However, we plan to explore the application of deep-learning features in larger datasets in the future.

In addition, we employed LASSO for feature selection due to its well-established use for analysis of high-dimensional datasets and its ability to avoid overfitting. Moreover, LASSO produces sparse models, and this helps to identify the most important of the many analyzed features. The advantages of LASSO are that it can simultaneously perform feature selection and regularization. By adding an L1 penalty to the regression model, LASSO increases sparsity, shrinks some coefficients to zero, and effectively selects a subset of the most relevant features. This makes it suitable for high-dimensional data where the number of features exceeds the number of samples. Other feature selection algorithms, such as Recursive Feature Elimination (RFE), iteratively remove features by training models, and this can be computationally intensive for large datasets. However, unlike LASSO, RFE does not have a built-in mechanism for penalizing model complexity. Random Forest Feature Importance ranks features based on their contribution to model ACC by using ensemble methods and can capture nonlinear relationships, but it does not inherently perform feature selection and the results may be less interpretable than those from LASSO. Based on these considerations, the main advantages of LASSO are its computational efficiency, interpretability, and ability to handle multicollinearity by selecting one feature from a set of correlated features.

However, this study has several limitations. First, despite the multicenter design, we only considered 92 patients overall and only 41 patients with RO. To address the small sample size, we divided the data into a training set and a validation set in a 7:3 ratio, ensured that the class distribution in the dataset was balanced, and combined our analysis with cross-validation techniques to minimize the risk of overfitting. Additionally, we are planning to collect additional data from other study centers for future studies, and then use these larger samples to validate the generalizability of models. Second, due to certain inconsistencies in the CT scanning protocols among the 5 centers, we excluded the excretory phase because this phase was not universally available. Third, we excluded unenhanced CT images because of their lower resolution, which could lead to bias and error during image segmentation. Finally, although we made efforts to minimize differences among CT scanners by use of resampling and normalization, some discrepancies may have persisted.

## Conclusion

This study successfully developed intratumoral and peritumoral radiomic models that reliably distinguished RO from CRCC using the CMP and NP of CT images. The CMP-based radiomic models were superior to the NP models and better than the experienced radiologists, highlighting their potential use for screening and diagnostic discrimination of RO and CRCC.

## Data Availability

The raw data supporting the conclusions of this article will be made available by the authors, without undue reservation.

## Supplementary material

Supplementary Figure 1 Importance weights of radiomics features in Model 4 **(A)**, Model 7 **(B)**, and Model 9 **(C)**.
