# Supplementary figures and images for: Intratumoral and peritumoral radiomics using multi-phase contrast-enhanced CT for diagnosis of renal oncocytoma and chromophobe renal cell carcinoma: a multicenter retrospective study
Source: Front Oncol. 2025 Feb 5;15:1501084. doi: 10.3389/fonc.2025.1501084 (PMC11835681; doi:10.3389/fonc.2025.1501084)

A

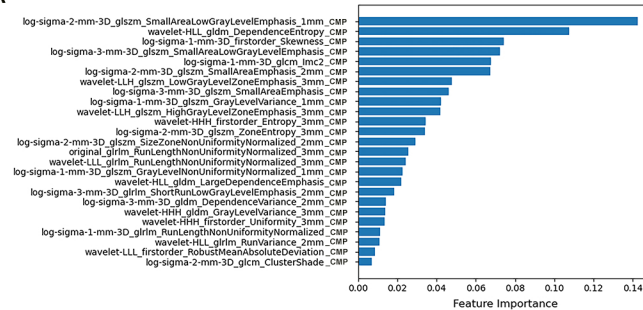

B

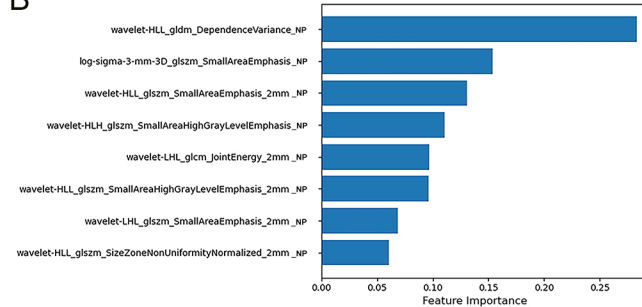

C

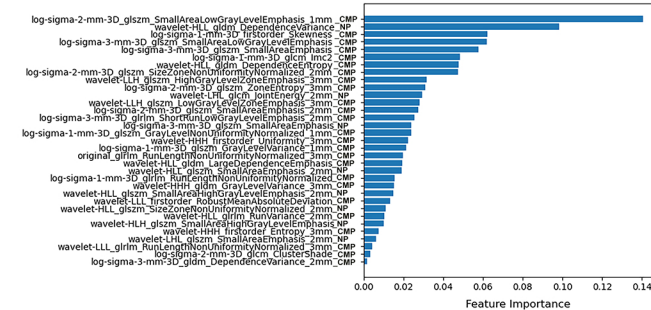

Supplement: Supplementary Figure 1 — Importance weights of radiomics features in Model 4 (A), Model 7 (B), and Model 9 (C). [file DataSheet1.pdf]
