# Supplementary material for: Intratumoral and peritumoral radiomics using multi-phase contrast-enhanced CT for diagnosis of renal oncocytoma and chromophobe renal cell carcinoma: a multicenter retrospective study
Source: Front Oncol. 2025 Feb 5;15:1501084. doi: 10.3389/fonc.2025.1501084 (PMC11835681; doi:10.3389/fonc.2025.1501084)
Supplement: Supplementary file 2 [file Table1.docx]

**Table S1.** Rad features of SVM classifiers used for analysis of CRCC and RO.

| ***Model 1* (9 features)** |
| --- |
| log-sigma-1-mm-3D_firstorder_Skewness_CMP |
| log-sigma-1-mm-3D_glcm_Imc2_CMP |
| log-sigma-1-mm-3D_glrlm_RunLengthNonUniformityNormalized_CMP |
| log-sigma-2-mm-3D_glcm_ClusterShade_CMP |
| log-sigma-3-mm-3D_glszm_SmallAreaEmphasis_CMP |
| log-sigma-3-mm-3D_glszm_SmallAreaLowGrayLevelEmphasis_CMP |
| wavelet-HLL_gldm_DependenceEntropy_CMP |
| wavelet-HLL_gldm_LargeDependenceEmphasis_CMP |
| wavelet-LLL_firstorder_RobustMeanAbsoluteDeviation_CMP |
| ***Model 2* (12 features)** |
| 9 Rad features from Model 1 |
| log-sigma-1-mm-3D_glszm_GrayLevelNonUniformityNormalized_1mm_CMP |
| log-sigma-1-mm-3D_glszm_GrayLevelVariance_1mm_CMP |
| log-sigma-2-mm-3D_glszm_SmallAreaLowGrayLevelEmphasis_1mm_CMP |
| ***Model 3* (17 features)** |
| 12 Rad features from Model 2 |
| log-sigma-2-mm-3D_glszm_SizeZoneNonUniformityNormalized_2mm_CMP |
| log-sigma-2-mm-3D_glszm_SmallAreaEmphasis_2mm_CMP |
| log-sigma-3-mm-3D_gldm_DependenceVariance_2mm_CMP |
| log-sigma-3-mm-3D_glrlm_ShortRunLowGrayLevelEmphasis_2mm_CMP |
| wavelet-HLL_glrlm_RunVariance_2mm_CMP |
| ***Model 4* (25 features)** |
| 17 Rad features from Model 3 |
| original_glrlm_RunLengthNonUniformityNormalized_3mm_CMP |
| log-sigma-2-mm-3D_glszm_ZoneEntropy_3mm_CMP |
| wavelet-LLH_glszm_HighGrayLevelZoneEmphasis_3mm_CMP |
| wavelet-LLH_glszm_LowGrayLevelZoneEmphasis_3mm_CMP |
| wavelet-HHH_firstorder_Entropy_3mm_CMP |
| wavelet-HHH_firstorder_Uniformity_3mm_CMP |
| wavelet-HHH_gldm_GrayLevelVariance_3mm_CMP |
| wavelet-LLL_glrlm_RunLengthNonUniformityNormalized_3mm_CMP |
| ***Model 5* (3 features)** |
| log-sigma-3-mm-3D_glszm_SmallAreaEmphasis_NP |
| wavelet-HLL_gldm_DependenceVariance_NP |
| wavelet-HLH_glszm_SmallAreaHighGrayLevelEmphasis_NP |
| ***Model 6*** |
| N/A |
| ***Model 7* (8 features)** |
| 3 Rad features from Model 5 |
| wavelet-LHL_glcm_JointEnergy_2mm_NP |
| wavelet-LHL_glszm_SmallAreaEmphasis_2mm_NP |
| wavelet-HLL_glszm_SizeZoneNonUniformityNormalized_2mm_NP |
| wavelet-HLL_glszm_SmallAreaEmphasis_2mm_NP |
| wavelet-HLL_glszm_SmallAreaHighGrayLevelEmphasis_2mm_NP |
| ***Model 8*** |
| N/A |
| ***Model 9 (33 features)*** |
| 33 Rad features from Model 4 (25) and Model 7 (8) |

CMP: Rad features from the corticomedullary phase; NP: Rad features from the nephrographic phase; 1mm, 2mm, and 3mm: Rad features for a peritumoral mask of 1 mm, 2 mm, and 3 mm

**Table S2**. P-values of DeLong test from pair-wise comparisons of ROC values of different models in the validation set.

|  | Model 1 | Model 2 | Model 3 | Model 4 | Model 5 | Model 7 | Model 9 |
| --- | --- | --- | --- | --- | --- | --- | --- |
| Model 1 |  | **0.02** | 0.56 | 0.19 | 0.60 | 0.49 | 0.46 |
| Model 2 | **0.02** |  | **0.01** | **0.02** | 0.34 | 0.43 | 0.17 |
| Model 3 | 0.56 | **0.01** |  | 0.09 | 0.37 | 0.27 | **0.02** |
| Model 4 | 0.19 | **0.02** | 0.09 |  | 0.17 | 0.12 | **<0.01** |
| Model 5 | 0.60 | 0.34 | 0.37 | 0.17 |  | 0.71 | 0.93 |
| Model 7 | 0.49 | 0.43 | 0.27 | 0.12 | 0.71 |  | 0.90 |
| Model 9 | 0.46 | 0.17 | **0.02** | **<0.01** | 0.93 | 0.90 |  |
